# Dietary Plasticity of African Clawless Otters ( *Aonyx capensis* ): An Assessment of Seasonal Variation in Prey Availability

**DOI:** 10.1002/ece3.71810

**Published:** 2025-07-15

**Authors:** Marli Burger, Andre Ganswindt, Tshepiso L. Majelantle, Juan Scheun, Andrea B. Webster

**Affiliations:** ^1^ Faculty of Natural and Agricultural Sciences Mammal Research Institute, University of Pretoria Pretoria South Africa; ^2^ Brain Function Research Group, Department of Physiology University of Witwatersrand Johannesburg South Africa; ^3^ Department Nature Conservation Tshwane University of Technology Pretoria South Africa

**Keywords:** African clawless otter, dietary flexibility, dominant prey item, insects, seasonal prey shifts

## Abstract

African clawless otters ( *Aonyx capensis* ) are opportunistic feeders with a broad dietary niche. Variation in their diet can be influenced by environmental and anthropogenic factors, which can affect seasonal and longitudinal prey availability. Flexibility in the diet allows African clawless otters to adapt to these changes and exploit novel prey items when available. Seasonal examination of otter spraints from three different locations across South Africa demonstrates that African clawless otters are able to shift from their preferred crab‐based diet to a fish‐ or insect‐based diet in response to environmental and anthropogenic drivers. Here we provide direct evidence of African clawless otters in terrestrial environments shifting to an insect‐dominated diet when this resource is available. The dietary plasticity and response of this species to the shifts in available prey items may be an important factor for future consideration in conservation management of the species.

## Graphical abstract

African clawless otters demonstrate dietary plasticity, but terrestrial insects have not previously been documented as a primary prey source in this species. Dietary flexibility and response to shifts in available prey items may offer African clawless otters an advantage in the face of climate change.

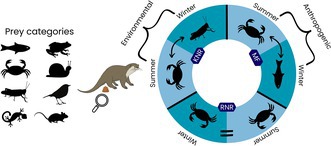

## 1 Introduction

In global terms, South Africa is a water‐limited country. Increasing pollution from anthropogenic sources and failing waste‐water treatment infrastructure have led to an overall decrease in water quality, with only 15% of South African rivers deemed to be in ‘good condition’ (Thirion and Jafta 2019). The dramatic increase in CO_2_ levels, higher temperatures, extended dry periods, and later, unpredictable rainfall patterns associated with climate change are likely to influence insect biology and ecology (Masson‐Delmotte et al. 2021). As temperature regulates insect physiology and metabolism, increasing temperatures combined with higher rainfall create ideal conditions for insect eggs (e.g., fall armyworm; *Spodoptera exempta* , and brown locust; *Locustana pardalina*) to hatch (Halubanza 2024). Additionally, higher temperatures increase insect physiology and activity, influencing metabolic rates and increasing resource requirements (Ma et al. 2021). Coupled with these drivers, elevated CO_2_ levels modify plant chemistry and decrease nutrition, increasing plant susceptibility to insectivorous herbivores (Johnson et al. 2020). Anthropogenic and environmental factors are therefore likely to influence biodiversity and subsequent seasonal prey availability in aquatic systems (Parmesan 2006; Spano et al. 2021).

South Africa is home to spotted‐necked otters ( *Hydrictis maculicollis* ) that feed primarily on fish and African clawless otters ( *Aonyx capensis* ), which feed primarily on freshwater crabs (*Potamonautes* spp.) (Somers and Nel 2007; Somers and Purves 1996). The opportunistic, generalist feeding habits of African clawless otters (hereafter referred to as otters) allow them to exploit a variety of other prey items, including fish, frogs, birds, molluscs and insects (Jordaan et al. 2015; Rowe‐Rowe and Somers 1998; Somers and Purves 1996). In some habitats where crustacean availability and accessibility are low, fish become the dominant prey item (Andarge et al. 2017; Watson and Lang 2003). This flexibility indicates that otters respond to seasonal changes in prey base, which can be influenced by environmental and anthropogenic disturbances (Jordaan et al. 2015; Somers 2000; Verwoerd 1987). Parasite burden is often linked to specific diets, and as a result, the spatio‐temporal variation in otter diet could lead to fluctuations in the consumption of endoparasites (Lutermann et al. 2014, 2015).

Human‐mediated and seasonal shifts in food availability can influence the dietary range of animals, significantly impacting species' behavior, movement patterns, fitness and survival (Stenset et al. 2016; Van Daele et al. 2012), which subsequently impacts the management and conservation of the species. To date, there is little evidence to show that African clawless otters make use of insects as a primary food source in addition to crustaceans and fish. Through the sampling of otter spraints, we aimed to investigate whether African clawless otters adjust their diet opportunistically to take advantage of novel prey item availability. Here we provide the first direct evidence that otters make use of terrestrial insects as a primary resource when available.

## 2 Methods

### 2.1 Study Sites

**FIGURE 1:**
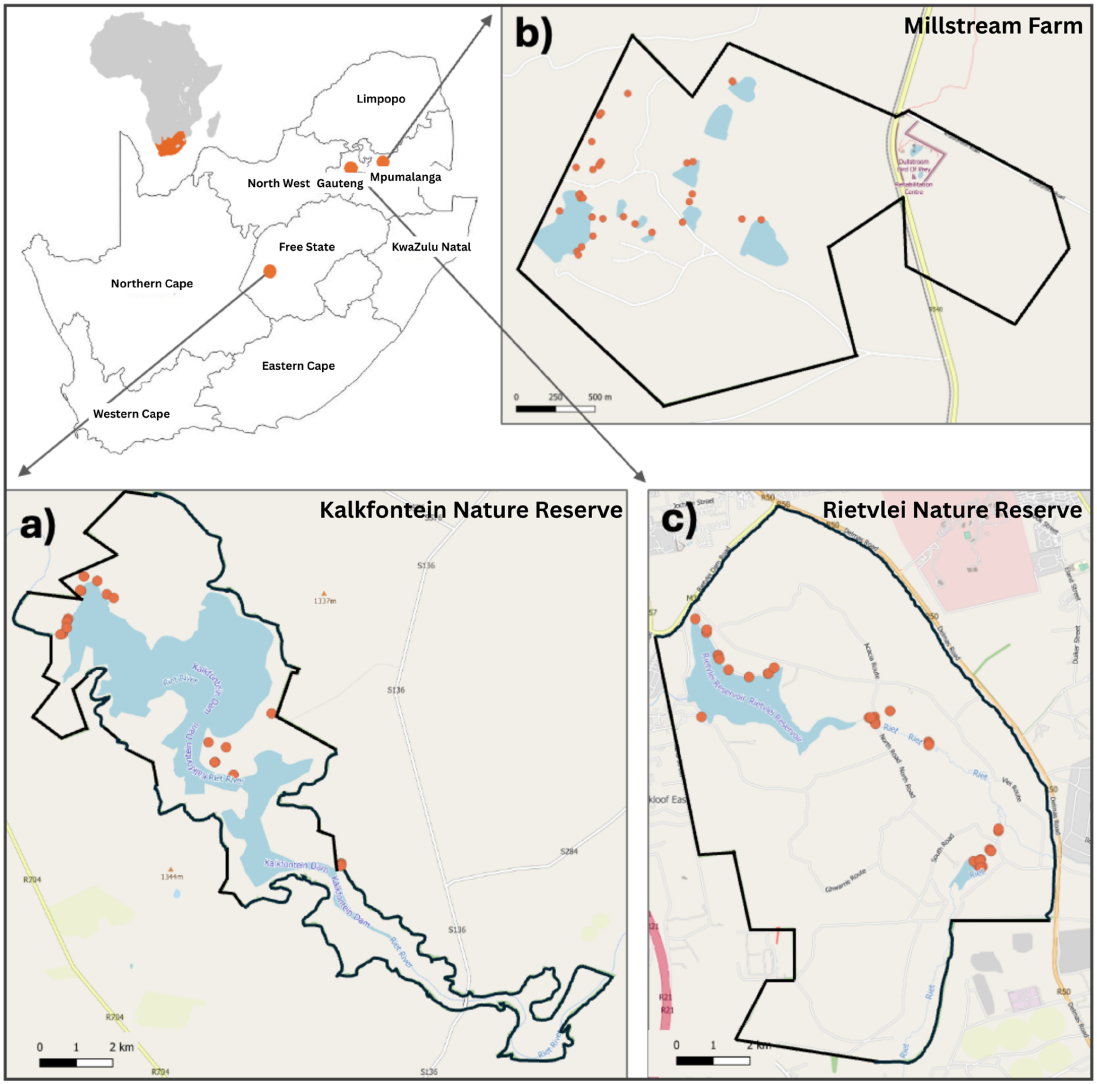
Continental orientation map and location of sampling sites across provinces in South Africa. Sampled Latrines (orange circles) are shown for (a) Kalkfontein Nature Reserve (KNR), Free State Province, (b) Millstream Farm (MF) Mpumalanga Province, and (c) Rietvlei Nature Reserve, Gauteng Province.

To investigate seasonal and spatial variation in African clawless otter diet, spraint (faecal matter) samples (*n* = 201) from three study sites across South Africa (Figure 1) were evaluated from July 2021 to January 2022. Kalkfontein Dam Nature Reserve (KNR) situated in the Free State province, supports a healthy, natural aquatic system that is home to a variety of birds, endemic plant species and fish (J. Josling, Kalkfontein Dam Nature Reserve Manager, personal communication). Millstream Farm (MF) situated in Mpumalanga province, is one of South Africa's leading fly‐fishing destinations and supports a variety of bird, mammal, fish and reptile species. Natural dams are connected to constructed trout ponds that are stocked with 90% rainbow and 10% brown trout ( *Salmo trutta* ) every 2 weeks (S. Vincent, Millstream Farm management, personal communication). Rietvlei Nature Reserve (RNR) is situated in the urban area of Gauteng province. The reserve supports a diverse range of mammal, bird, fish and reptile species. Sites were chosen based on previous observations of African clawless otters (Majelantle et al. 2020; Majelantle et al. 2021; Jordaan et al. 2021; S. Vincent, Millstream Farm management, personal communication; M. Somers (previous research at RNR), personal communication; J. Josling, Kalkfontein Dam Nature Reserve management, personal communication). Site orientation map was created using OpenStreetMap (https://www.openstreetmap.org/#map=9/‐29.712/24.521) in QGIS version 3.34.3.

### 2.2 Sample Identification and Collection

African clawless otter spraint samples were collected from latrines visited daily between 6 :00 and 9:00 h around each site during the dry winter (May–September, *n* = 122 from 16 latrines) and wet summer (October–April, *n* = 80 samples from 17 latrines) periods (Table 1). Spraint samples were identified based on physical characteristics including size (diameter = ca. 25 mm), shape, characteristic smell and tracks surrounding latrines (Libenberg 1990; Rowe‐Rowe 1977). On occasion, spraints were collected after otters were seen defaecating at specific locations; however, the majority of samples were collected using the above criteria from previously identified latrine sites. Anal jelly texture, temperature and moisture content were used to determine sample freshness. As latrines are used for olfactory communication between individuals, each faecal deposit was subsampled to minimise communication disturbance. To avoid contamination by substrate, subsamples were collected from the centre of the faecal pile using disposable gloves. Samples were placed into individually labelled plastic screw top containers and kept cool in a portable cooler box with ice bricks until return to base camp within a maximum of 3 h. Samples were stored at −20°C and transported by road to the University of Pretoria's Endocrine Research Laboratory until further processing and analysis.

**TABLE 1:** Spraint samples (*n* = 201) collected from various latrines at three different study sites, between two seasons.

Site	Season	Latrines	Samples
Kalkfontein Nature Reserve	Dry: 5 July–11 July 2021	5	Dry: *n* = 34
	Wet: 5 January–12 January 2022	4	Wet: *n* = 19
Millstream Farm	Dry: 22 July–30 July 2021	5	Dry: *n* = 65
	Wet: 3 November–9 November 2021	6	Wet: *n* = 39
Rietvlei Nature Reserve	Dry: 29 July–13 August 2021	6	Dry: *n* = 22
	Wet: 22 November–30 November 2021	7	Wet: *n* = 22

### 2.3 Diet Analysis

Spraint samples were individually lyophilized, visually inspected and sorted into crab, fish, mammals, insects, amphibians, reptiles, birds, molluscs, unidentifiable material and non‐food item categories based on morphological characteristics of macro components (Somers and Purves 1996) for later comparison between sites. Vertebrates (fish, mammals, amphibians, reptiles and birds) were identified by means of skeletal remains together with distinctive keratin structures such as scales, hair, feathers, beaks, nails, talons, or claws and on occasion, undigested skin. Exoskeletons were used to identify crustaceans and invertebrates (crabs, insects and molluscs; Somers and Purves 1996). Only brown locusts were identified to species level as they were intact in the fecal material after lyophilization. The majority of fecal content could be categorized with the naked eye, but prey items such as insect remains were classified using a Vickers light microscope at ×4/0.10 or ×10/0.25 magnification, depending on the size of the item.

### 2.4 Data Analysis

To obtain a reliable indication of the importance of different food items at each site and determine and compare overall differences in the diet of otters between sites and seasons, the relative percentage of occurrence (RPO) and percentage of occurrence (PO) were calculated (Erlinge 1968) using the following equations:

$$\mathrm{RPO}=\frac{\text{Total number of occurrences of}\;a\;\text{specific prey item in}\;\mathrm{all}\;\text{of the faecal samples}}{\text{Total number of occurrences of}\;\mathrm{all}\;\text{prey items recorded in}\;\mathrm{all}\;\text{of the faecal samples}}\times 100$$

The RPO represents the percentage of a specific prey item in relation to the total number of prey items consumed by the population (Crowley et al. 2018; Rowe‐Rowe and Somers 1998).

$$\mathrm{PO}=\frac{\text{Number of faecal samples containing}\;a\;\text{specific prey item}}{\text{Total number of faecal samples}}\times 100$$

The PO indicates the percentage of samples containing a specific prey item (Crowley et al. 2018; Rowe‐Rowe and Somers 1998).

To evaluate and compare the spatial and temporal differences in otter diet, variation in RPO and PO was calculated seasonally for each prey item at respective study sites (Crowley et al. 2018; Rowe‐Rowe and Somers 1998). Percentage differences between sites and season were compared using descriptive statistics. To assess the difference in PO within sites relative to season (e.g., KNRw vs. KNRs), and across sites within the same season (e.g., KNRw vs. MFw vs. RNRw), a chi‐squared test with a Yates correction was used for each prey item, following the methodology outlined in Jordaan et al. (2015). To account for multiple comparisons across site and season combinations, we applied a Bonferroni correction to the *p*‐values obtained from Chi‐square tests. All statistical analyses were carried out using the stats package in the R programming language (version 4.4.1; R Core Team 2022) on the RStudio IDE (version 2024.4.2.764; Posit Team 2024), with the level of significance set at *p* ≤ 0.05.

## 3 Results

### 3.1 RPO

**FIGURE 2:**
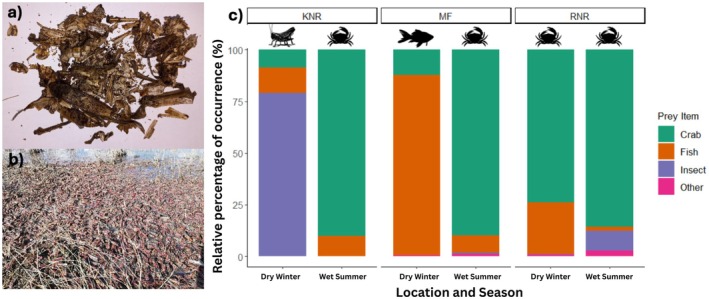
(a) Lyophilised otter spraint from Kalkfontein Nature Reserve contains only brown locust remains. (b) Brown locusts accumulate at the water's edge at Kalkfontein Nature Reserve. (c) Bar graph indicating the relative percentage of occurrence (RPO; %; *y*‐axis) of different prey items identified in 201 spraints collected from free‐ranging African clawless otters between wet and dry seasons (*x*‐axis) and across the three different study sites (KNR, Kalkfontein Nature Reserve; MF, Millstream Farm; RNR, Rietvlei Nature Reserve). The remaining prey items labelled as ‘other’ (mammal, amphibian, reptile, bird and mollusc) contributed < 3% to the overall RPO.

Based on the RPO otters at KNR, predominantly ate brown locusts during the winter (79%; *n* = 34) and crabs during the summer (90%; *n* = 19). At MF, otters fed predominantly on trout during the winter (90%; *n* = 65) and crabs during the summer (90%; *n* = 39) while at RNR, otters fed primarily on crabs during both winter (74%; *n* = 22) and summer (86%; *n* = 22; Figure 2a–c).

### 3.2 PO for Each Prey Species

The PO of crab, fish, insect, mammal and reptile prey categories was significantly different (*p* < 0.05) within and between study sites and between the wet and dry season (Table 2).

**TABLE 2:** A summary of the percentage of occurrence (PO) of all the prey items detected (*n* = 201) in African clawless otter spraints collected from the three study sites.

Season			Prey item							
			Crab	Fish	Mammals	Insects	Amphibian	Reptile	Bird	Mollusc
Winter	KNR	*n*	8	18	0	34	1	0	1	0
	(*n* = 34)	PO	23.53	52.94	0.00	100.00	2.94	0.00	2.94	0.00
		*p* < 0.05	ab*	ab	a	ab*		ab		
	MF	*n*	55	60	2	8	2	13	0	0
	(*n* = 65)	PO	84.62	92.32	3.08	12.31	3.08	20.00	0.00	0.00
		*p* < 0.05	a	a	b	a		*a		
	RNR	*n*	23		4	2	0	2	0	0
	(*n* = 22)	PO	100.00	90.92	13.64	9.09	0.00	9.09	0.00	0.00
		*p* < 0.05	b	b*	ab	b*		*b		
Summer	KNR	*n*	17		0	1	0	0	0	0
	(*n* = 19)	PO	89.47	31.59	0.00	5.26	0.00	0.00	0.00	0.00
		*p* < 0.05	*	c	c	ce*				
	MF	*n*	39	25	2	9	0	0	0	0
	(*n* = 39)	PO	100.00	64.10	5.13	23.08	0.00	0.00	0.00	0.00
		*p* < 0.05		c		c		*		
	RNR	*n*	22	9	2	9	0	0	1	1
	(*n* = 22)	PO	100.00	40.91	9.09	40.91	0.00	0.00	4.55	4.55
		*p* < 0.05		*	c	e*		*		

*Note:* Chi‐squared tests of association, with a Yates' correction were performed to test for significant inter‐site (i.e., within a single season across sites—KNRw vs. MFw vs. RNRw) and inter‐seasonal (within a single site across seasons—KNRw vs. KNRs) differences within a prey category. To account for multiple comparisons across site and season combinations, we applied a Bonferroni correction to the *p*‐values obtained from chi‐squared tests. The asterisk (*) indicate significant inter‐seasonal differences (*p* < 0.05) within the prey category, while the corresponding letters (top to bottom) indicate significant inter‐site differences (*p* < 0.05) within the prey category.

## 4 Discussion

Overall, there was seasonal variation in dominant prey items utilized at two of the three study sites. The PO differed significantly across study sites for crab, fish, insect, mammal and reptile, with differences between certain site‐season pairs. Our findings align with previous research on African clawless otter diet (Andarge et al. 2017; Jordaan et al. 2015; Rowe‐Rowe 1977; Somers and Purves 1996; Watson and Lang 2003) that suggest African clawless otters have a broad dietary niche. Results from this study highlight crab as being the most important prey item at all three study sites during the wet summer season, but that African clawless otters vary their diet during the dry winter season across all three study sites. At RNR, crab remained the main prey item during the dry season, while insects and fish were the main prey items at KNR and MF, respectively. Temporal and spatial variation in otter diet could be the result of vegetation cover, climate, water temperature, anthropogenic disturbance and prey base availability (Andarge et al. 2017; Jordaan et al. 2015; Somers 2000; Verwoerd 1987).

Previous research highlights the unusual importance of insects (specifically dobsonfly larvae; Insecta, Megaloptera) as a dietary item when abundant for the Neotropical otter (*Lontra annectens*; Pozos‐López et al. 2024), and aquatic insects have been identified as the third most common prey type for the Eurasian otter ( *Lutra lutra* ; Taylor et al. 2010). Although insects are known to form a small part of the African clawless otters' diet, there is no record of terrestrial insects, specifically brown locusts, being the main prey item of this species in previous studies (Andarge et al. 2017; Jordaan et al. 2015; Rowe‐Rowe 1977; Somers and Purves 1996; Watson and Lang 2003). During the winter sampling period (July 2021), brown locust (*Locustana pardalina*) outbreaks occurred in many provinces across South Africa, including the Free State (M. Burger, personal observation), which were reflected in the diet of otters at KNR during this time. The utilization of terrestrial insects by otters at the KNR site is a novel finding and supports the theory that African clawless otters are opportunistic feeders and able to adapt to and exploit novel prey items within their habitat to meet their energy requirements (Andarge et al. 2017; Jordaan et al. 2015; Somers 2000; Verwoerd 1987).

At MF, during the summer months, anthropogenic stocking of dams with supplement‐fed trout (90% rainbow and 10% brown trout) occurs every 14 days (S. Vincent, Millstream Farm management, personal communication). However, in the colder winter months, anthropogenic stocking of supplement‐fed trout increases to once every 9 days. Otter spraints from this site reflect an increase in fish consumption coupled with higher parasite loads in a small percentage (13.5%) of samples during the dry winter season, which can be linked to the increase in trout supplementation over this period. In addition, crabs are inactive over this period, finding refuge in small crevices up to 1 m deep, making them difficult for predators such as otters to obtain (Rowe‐Rowe 1977). Results from this site indicate that due to crab inaccessibility, otters adapt their diet during winter to benefit from the increased availability of trout.

The dietary plasticity of African clawless otters allows them to exploit multiple species to meet their dietary requirements. However, consumption of farmed fish can result in a higher parasite load, which can compromise overall health. Due to the higher stocking densities in aquaculture systems, farmed trout generally carry a higher parasite load than wild trout. Higher stocking densities result in closer proximity between individuals, facilitating the rapid spread of parasites between individuals within the population (Krkošek 2017; Lafferty et al. 2015). Additionally, limited space and the potential stress of confinement may influence immune system function (Palme 2019), further contributing to an environment that promotes parasite proliferation (Krkošek 2017; Lafferty et al. 2015). Although otters at MF have access to a high‐quality prey resource, consumption of farmed fish comes with significant costs and potential health risks.

While seasonal variation and a shift to a fish dominated diet have been observed in previous studies of African clawless otters (Andarge et al. 2017; Jordaan et al. 2015; Somers 2000), the shift to a diet dominated by terrestrial insects has previously not been recorded. Despite the relatively small sample sizes obtained due to low density of otters in this study, our results reflect biologically relevant differences in seasonal feeding habits. This flexibility supports the notion that otters exhibit dietary plasticity driven by opportunistic feeding behaviour. Considering that freshwater crabs (*Potamonautes* sp., Dalu et al. 2016) and endemic fish species (Nyboer et al. 2019) are vulnerable to the effects of climate change, and locust outbreaks are predicted to increase, especially in regions where African clawless otters naturally occur (Peng et al. 2020), insects are likely to become an important part of the otter diet in response to climate change and fluctuations in environmental conditions. Although otters utilize both anthropogenic and environmentally driven food resources, the reliance on farmed trout presents potential ecological and health consequences, while an increasing availability of locusts may offer a high‐quality but fluctuating alternative. Otters' response to changing environmental factors and biodiversity shifts in aquatic systems, particularly their ability to exploit novel dietary resources, may require a reassessment of current conservation and management strategies.

## Author Contributions

**Marli Burger:** conceptualization (lead), data curation (lead), formal analysis (lead), investigation (lead), methodology (equal), project administration (lead), visualization (equal), writing – original draft (lead). **Andre Ganswindt:** conceptualization (equal), funding acquisition (lead), supervision (equal), validation (lead), writing – review and editing (supporting). **Tshepiso L. Majelantle:** conceptualization (equal), formal analysis (equal), supervision (equal), visualization (equal), writing – review and editing (supporting). **Juan Scheun:** supervision (supporting), writing – review and editing (supporting). **Andrea B. Webster:** conceptualization (equal), project administration (supporting), supervision (equal), visualization (supporting), writing – review and editing (lead).

## Ethics Statement

This study was conducted with the approval of the University of Pretoria's Research and Animals Ethics and Care Committees (NAS087/2020) in compliance with animal diseases from the Department of Agriculture, Land Reform and Rural Development (DALRRD; section 20; SDAH‐Epi‐21110813130).

## Conflicts of Interest

The authors declare no conflicts of interest.

## Data Availability

Data is available at https://doi.org/10.25403/UPresearchdata.24324880.v1.
